# The use of a functional test battery as a non-invasive method of fatigue assessment

**DOI:** 10.1371/journal.pone.0212870

**Published:** 2019-02-28

**Authors:** Steven Hughes, Dale W. Chapman, G. Gregory Haff, Sophia Nimphius

**Affiliations:** 1 Physiology Discipline, Australian Institute of Sport, Bruce, ACT, Australia; 2 Centre for Exercise and Sports Science Research, School of Medical and Health Sciences, Edith Cowan University, Joondalup, WA, Australia; University of Tennessee Health Science Center College of Graduate Health Sciences, UNITED STATES

## Abstract

To assess whether a battery of performance markers, both individually and as group, would be sensitive to fatigue, a within group random cross-over design compared multiple variables during seated control and fatigue (repeated sprint cycling) conditions. Thirty-two physically active participants completed a neuromuscular fatigue questionnaire, Stroop task, postural sway, squat jump, countermovement jump, isometric mid-thigh pull and 10 s maximal sprint cycle (Sprint^max^) before and after each condition (15 min, 1 h, 24 h and 48 h). In comparison to control, larger neuromuscular fatigue questionnaire total score decrements were observed 15 min (5.20 ± 4.6), 1 h (3.33 ± 3.9) and 24 h (1.83 ± 4.8) after cycling. Similarly, the fatigue condition elicited greater declines than control at 15 min and 1 h post in countermovement jump height (1.67 ± 1.90 cm and 1.04 ± 2.10 cm), flight time-contraction time ratio (0.03 ± 0.06 and 0.05 ± 0.11), and velocity (0.06 ± 0.07 m∙s^-1^ and 0.04 ± 0.08 m∙s^-1^). After fatigue, decrements were observed up to 48 h for average Sprint^max^ cadence (4–6 RPM), up to 24 h in peak Sprint^max^ cadence (2–5 RPM) and up to 1 h in average and peak Sprint^max^ power (45 ± 60 W and 58 ± 71 W). Modelling variables in a stepwise regression demonstrated that CMJ height explained 53.2% and 51.7% of 24 h and 48 h Sprint^max^ average power output. Based upon these data, the fatigue induced by repeated sprint cycling coincided with changes in the perception of fatigue and markers of performance during countermovement and squat jumps. Furthermore, multiple regression modelling revealed that a single variable (countermovement jump height) explained average power output.

## Introduction

The ability to effectively monitor fatigue is highly sought after by coaches and exercise scientists of elite athletes. Classically, neuromuscular fatigue has been defined as an acute reduction in task performance which includes both an increased perceived effort to exert force as well as an eventual inability to produce force [1]. In sport, fatigue would manifest as a reduction in the ability to perform the desired movement, exercise, or skill and may encompass metabolic and/or neuromuscular and/or cognitive fatigue. In high performance sport a number of performance markers are often used to assess fatigue such as perceptual questionnaires [2, 3], jump tests [2, 4], maximal and submaximal sprints [2], heart rate variables [2, 3], hormone levels [5] and postural sway measurements [4].

The capacity to effectively monitor athlete fatigue provides coaches and scientists with the ability to better understand resistance training and conditioning periodisation, as well as the adaptation and tapering response. A deeper understanding of periodisation, adaptation, and tapering allows improved application to the processes typically employed to optimise training or improve competition performance. However, despite a wealth of research in the area of athletic fatigue, single performance markers have rarely been definitive in all situations [6–8] suggesting that no one marker is able to truly reflect fatigue status. This is likely due to the nature of fatigue where the underpinning mechanisms are dependent on the type of task performed and confounded by participant motivation, psychological status, muscle activation pattern, intensity, duration, and the continuous or intermittent nature of the task [9]. For this reason perhaps a new approach to the utilization of performance markers is needed. Though research has focused on single [10, 11] or multiple markers as a measure of fatigue [4, 8, 12], rarely has research on multiple performance markers utilised a modelling approach to explain fatigue. The multifactorial nature of fatigue may suggest that a single all-encompassing test to measure fatigue may not exist. Therefore, the present investigation sought to assess whether a range of performance markers would decline in response to a high intensity sprint cycle fatigue protocol at a number of time points up to 48 h post fatigue inducement. The second aim of this research was to assess whether a stepwise regression analysis of performance markers would explain decrements in 10 s maximal sprint (Sprint^max^) cycle performance.

Performance changes in healthy, recreationally active participants were measured before and after a fatiguing protocol by utilising a variety of independent performance markers reflective of the multifactorial nature and origin of fatigue. Performance markers aimed to assess internal perceptions of fatigue using a standard questionnaire, cognitive function through the Stroop task and aspects of the central nervous systems autonomous control functions through postural sway tasks. Furthermore lower limb movement capacity, strength and power performance was assessed using jump and isometric strength tests. Multiple tasks were chosen based on a range of body functions reported to be sensitive to fatigue [3, 4, 13] in response to many forms of exercise with the hypothesis that a broad range of tests would therefore be more likely to increase sensitivity to a large range of fatigue inducements. After identifying the most responsive variables to a fatiguing task, their capacity to elucidate the fitness-fatigue relationship was modelled. The outcomes from this work may help to inform coaches and sports scientists on predicting and understanding an athlete’s readiness for the next training stimuli.

## Methods

A within group random cross-over design was used to compare the fatigue test battery across multiple time points and assess the sensitivity of performance markers to a randomly assigned seated control and repeated sprint cycle exercise condition.

### Participants

Thirty-two physically active participants (24 males: 27.8 ± 7.6 years; 81.4 ± 11.1 kg and 8 females: 24.5 ± 3.5 years; 69.0 ± 14.1 kg) were recruited from sport and health science courses at local institutions. Each participant completed all testing sessions in the study. Participants were familiar with resistance exercise with a history of at least 6 months general resistance training. Participants were asked to avoid any strenuous exercise 24 h prior to testing and during the 24 h and 48 h follow up assessment period. A period of at least 24 h separated the familiarisation, control and fatigue blocks. Each participant was provided with a standardised meal (CHO = 1–1.5 g∙kg^-1^; Protein = 0.3 g∙kg^-1^, Fat = 0.28–0.47 g∙kg^-1^) that was consumed 2 h prior to all testing sessions and a 600 ml bottle (618 kJ) of Gatorade sports drink (PepsiCo, New York, USA) during testing sessions. Participants were instructed to avoid consumption of coffee prior to testing sessions and were requested to maintain their current nutrition intake throughout the study.

Participants attended seven laboratory testing sessions, a familiarisation session and three sessions each for control and fatigue conditions. Bike setup, isometric mid-thigh pull (IMTP) height assessment and wrist wrap familiarity were completed during the familiarisation session to ensure correct testing procedures throughout each of the six sessions.

**Fig 1:**
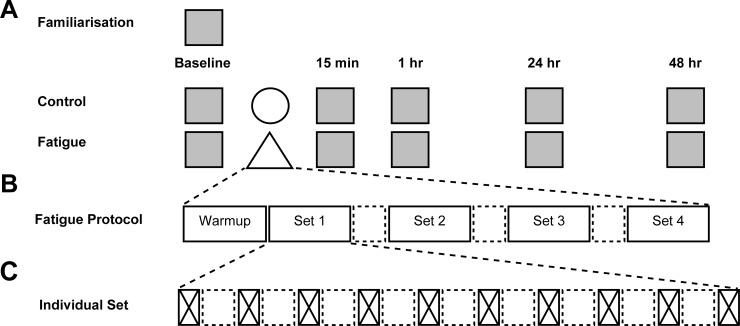
Study protocol overview. (A) Fatigue test battery testing (grey squares) with control protocol (circle) and fatigue intervention (triangle). (B) Fatigue intervention consisting of 4 sprint sets interspersed with 90 s active recovery (dashed square). (C) Sprint set breakdown consisting of 10 x 6 s sprints (crossed rectangle) with 30 s active recovery (dashed square).

For both conditions, participants completed baseline performance marker assessments followed by the intervention or control. Following this, participants completed performance marker assessments at multiple time points (15 min, 1 h, 24 h and 48 h) post intervention (control or fatigue) (Fig 1). The six performance tests were completed by each participant in a consistent order with the order set as least to most metabolic exertion [14]; neuromuscular fatigue questionnaire (NFQ), Stroop task, postural sway, squat jump (SJ), counter movement jump (CMJ), IMTP and 10 s maximal sprint cycle.

The study was approved by the Edith Cowan University Human Research Ethics Committee (Approval # 16284). Each participant was provided a written outline of the study requirements and given verbal instructions on how to perform all testing and exercise tasks. After an opportunity to answer any specific questions, written informed consent was obtained from all participants.

### Procedures

#### Fatigue protocol

A previously used repeat sprint protocol [15, 16] was modified (see Fig 1B) and performed on a Wattbike cycle ergometer (Nottingham, UK–Version 2.50.49). The modified protocol consisted of 4 sets of 10 x 6 s maximal sprints with a 30 s active recovery between sprints and an additional 90 s active recovery between sets. With the addition of a 5 min warmup, the protocol duration was 33.5 min with a combined sprint time of 4 min and 24.5 min of active recovery.

#### Fatigue test battery

A standardised warmup was completed on each testing day consisting of 7 min self-selected intensity cycling, 10 x 3 kg medicine ball squat/shoulder press, 10 x 3 kg medicine ball chest pass, 6 x bodyweight squats and 6 x bodyweight CMJs.

#### Neuromuscular fatigue questionnaire

An adaptation of the “individualised neuromuscular quality of life” questionnaire [17] was used to assess perceptual fatigue and functioning of participants. The adapted questionnaire (See S2 File. Neuromuscular Fatigue Questionnaire) encompassed four questions answered using a 7-point Likert scale to rate levels of tiredness/general fatigue, specific muscular fatigue, pain as a result of muscular fatigue as well as difficulty performing everyday tasks. The Likert scale utilised a rating from 0 to 6 with 0 being “not at all” and 6 being “an extreme amount”.

#### Stroop task

The Stroop task used to assess cognitive status consisted of two sets of 30 PowerPoint (Microsoft, USA) slides (6 colours x 5 slides) displayed in incongruent ink colours. Participants were required to articulate the ink colour not the written word with an exception to this rule if the ink was displayed in red [18]. Each slide progressed only after a correct answer with the total time taken to complete the 30 slides recorded. Chosen sets were assigned randomly from five sets to prevent potential learning effects.

#### Postural sway

Participants performed a 30 s barefoot static postural balance task on a dual force platform (9286BA, Kistler, Winterthur, Switzerland) positioned 0.35 m in front of a blank white wall to remove visual reference points. All 30 s data collections were completed using MARS software (2875A, Kistler, Winterthur, Switzerland) and sampled at 1000 Hz. Participants were positioned with a base of support of hallux and fifth metatarsal head 0.10 m apart and performed 3 x 30 s of quiet standing, one in each of the following conditions; head facing forward with neutral neck position and eyes open, head facing forward neutral neck position and eyes closed and eyes closed head back (30° in the sagittal plane). A seated rest period of 45 s was given between trials with the trials completed in order of task (postural control) difficulty. Total sway path length, total sway velocity and the area of 100% ellipse were calculated for each of the three conditions. MARS software defined the total sway path length (mm) as the combined trajectory of centre of pressure and calculated the sway velocity (mm∙s^-1^) by dividing the total sway path length by the collection duration. Finally the area of the 100% ellipse (mm^2^) was defined as the area of the ellipse fitted over the centre of pressure trajectory so that it contained 100% of the data points.

#### Squat jump, counter movement jump and isometric mid-thigh pull

Three separate muscle function tests; concentric-only SJ, CMJ and an IMTP were completed using dual force platforms (9286BA, Kistler, Winterthur, Switzerland) sampling at 1000 Hz.

Participants completed two sets of three body weight SJ and CMJ with a lightweight (0.4 kg) aluminium bar held across the shoulders. Instructions to jump as high as possible while “pushing off the ground as hard and as fast as possible” were provided. Between repetitions, 10 s rest was given with 45 s rest periods between each set. For the SJ, participants maintained an isometric squat with a self-selected 90° knee bend for 3 s before jumping vertically. Any trials with an eccentric force dip >5% were discarded and repeated. The CMJ consisted of a counter movement before a maximal vertical jump.

The IMTP protocol required participants on a force plate to pull as hard as possible for 3 s on an immovable bar (25 mm diameter; 460 MPa [min] Tensile Strength; 370 MPa [min] Yield Stress). The bar was fixed in a customised power rack (Crossrig, Aussie Strength Equipment, Australia). Body positioning was set as described by Haff et al. [19] with the bar height recorded and replicated for each testing session. Wrist straps were used to ensure maximal grip with external focus instructions to “push the ground as hard and as fast as you possibly can” [20] provided to participants. Two repetitions were performed separated by 2 min rest with a third performed if a 200 N difference was seen between the peak force of the two efforts.

Recording and calculation of variables were completed using Templo Jump Analysis software (Version 2016.1.404 Contemplas GmbH, Kempten, Germany) with SJ and CMJ variables of peak velocity (m∙s^-1^) and peak jump height (cm) calculated from ground reaction force traces, and relative forces (N∙kg^-1^) of SJ, CMJ and IMTP calculated using peak ground reaction force and bodyweight. Flight time to contraction time ratio (FT:CT) was calculated for SJ and CMJ with the contraction time represented as the time difference between the subject leaving the force plate and the concentric contraction of SJ or initiation of countermovement in CMJ [21].

#### 10 s sprint cycle

The Sprint^max^ was completed on a high moment of inertia flywheel, air-braked cycle ergometer (AIS, Australia). Power output was recorded at the ergometer cranks using a scientific version (8 strain-gauge) SRM power meter (Schoberer Rad Meßtechnik, Germany) at a sampling rate of 2 Hz. The SRM power meter was calibrated dynamically prior to testing. The ergometer seat and handlebar position was positioned individually for each participant and consistent across all sessions.

### Data analysis

Visual inspection of Q-Q plots was used to assess normality of data. Data were analysed in SPSS (v 19.0 IBM, New York, NY) using two-way (condition × time) repeated-measures ANOVA and a one-way repeated-measures ANOVA to analyse simple main effects of condition and time where an interaction effect was seen. A Greenhouse-Geisser correction was used where sphericity was violated. Variables used in analysis were total NFQ score, Stroop task duration, postural sway path, postural sway velocity and postural sway area of 100% ellipse, SJ and CMJ relative peak force, FT:CT, peak velocity and jump height, IMTP relative peak force, Sprint^max^ average cadence, peak cadence, average power and peak power. Multiple trials at each time point were averaged for Stroop task, SJ, CMJ, IMTP and Sprint^max^. Significance was set at α = 0.05 and the Benjamini-Hochberg procedure was employed to correct for multiple comparisons and decrease false discovery rates. Two-way interaction results are presented with p value and Cohen’s effect size (d).

Where two-way interactions were observed, correlation coefficient analysis was completed. Variables with <0.8 correlation were entered into a stepwise regression model to assess whether individual or grouped test battery variable changes could explain the change in Sprint^max^ average power. Significance for the stepwise regression was set at α = 0.05. Independence of residuals was assessed by the Durbin-Watson test and homoscedasticity was assessed by visual inspection of a plot of studentized residuals versus unstandardized predicted values.

Cohen’s effect sizes for two-way repeated-measures ANOVA and stepwise regression were calculated from partial eta squared and Cohen’s f^2^ values to present uniform effect sizes [22]. The magnitude of effect sizes were classified as trivial (<0.19), small (0.2–0.49) medium (0.5–0.79) large (>0.8).

## Results

One participant was removed due to competitive sport participation <24 h before baseline resulting in 31 participants. Due to equipment malfunction, data for a single subject was not collected for NFQ (1 time point) and Sprint^max^ (2 time points). Participants consumed all meals and consumption of Gatorade was consistent for each participant across sessions. The mean power decrement between the first sprint in the fatigue inducement cycle and subsequent set averages were 25%, 29% and 29% for set 2, 3 and 4.

No significant two way interactions were observed in SJ height (p = 0.065, d = 0.62), Stroop task (p = 0.187, d = 0.47), or relative forces for CMJ (p = 0.150, d = 0.50), SJ (p = 0.054, d = 0.66), and IMTP (p = 0.622, d = 0.29). Postural sway measured in eyes open, eyes closed or eyes closed head back tasks did not differ significantly with no interaction observed in metrics of sway path (p = 0.381, d = 0.37; p = 0.171, d = 0.47; p = 0.208, d = 0.44 respectively) velocity (p = 0.379, d = 0.37; p = 0.186, d = 0.47; p = 0.205, d = 0.45 respectively), and area of 100% ellipse (p = 0.607, d = 0.28; p = 0.568, d = 0.27; p = 0.114, d = 0.51 respectively). Simple main effect analysis revealed no significant differences between fatigue and control baseline measurements for any variables.

**Fig 2:**
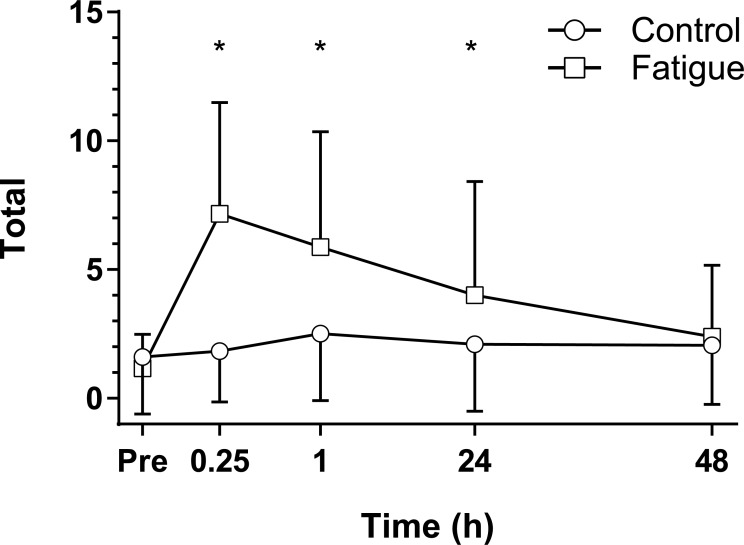
Neuromuscular fatigue questionnaire results. The mean (SD) neuromuscular fatigue questionnaire responses (n = 30) for the control (circles) and fatigue interventions (squares) from immediately prior (Pre) to 48 h post condition. *Significant (p < 0.05) difference between conditions at the identified time point using a Benjamini-Hochberg post hoc procedure.

At baseline, total NFQ score was 1.6 ± 2.2 and 1.2 ± 1.3 for control and fatigue respectively (Fig 2). A two-way interaction was seen between condition and time for NFQ (p < 0.0005, d = 1.74). A non-significant peak increase of 50% was seen 1 h post control condition and remained 28% greater at 48 h post. In comparison the fatigue condition significantly increased 489%, 386%, and 233% at 15 min (p < 0.0005, d = 1.88), 1 h (p < 0.0005, d = 1.43) and 24 h (p = 0.011, d = 0.87) post intervention respectively. A 100% increase between baseline and 48 h post intervention in the fatigue condition was not significant despite a medium effect size (d = 0.57). Significant differences between control and fatigue intervention were observed at 15 min (p < 0.0005, d = 1.58), 1 h (p < 0.0005, d = 0.92) and 24 h (p = 0.034, d = 0.53) time points.

**Fig 3:**
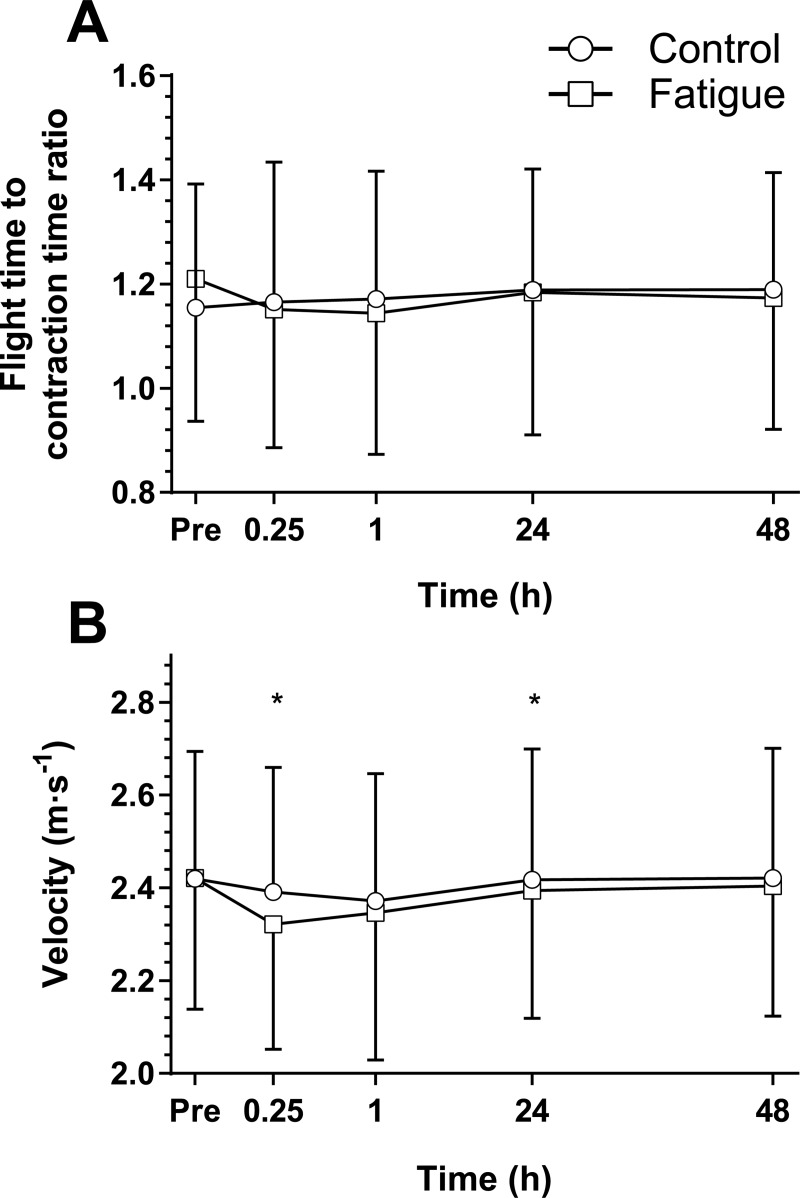
Squat jump results. The mean (SD) squat jump result (n = 31) for the control (circles) and fatigue interventions (squares) of (A) flight time to contraction time ratio (FT:CT) and (B) peak propulsive (concentric) velocity. Time points from immediately prior (Pre) to 48 h post condition. *Significant (p < 0.05) difference between conditions at the identified time point using a Benjamini-Hochberg post hoc procedure.

The SJ velocity at baseline was 2.42 ± 0.27 m∙s^-1^ and 2.42 ± 0.28 m∙s^-1^ in control and fatigue respectively (Fig 3) with a two way interaction present (p = 0.033, d = 0.67). Control SJ velocity had a significant decrease of 1% at 15 min (p = 0.004, d = 0.11) post control with a peak decrease of 2% occurring at 1 h (p = 0.003, d = 0.18) before returning to baseline. In response to the intervention, the fatigue condition showed a peak decrease of 4% at 15 min (p < 0.0005, d = 0.36) post intervention with a decrease of 3% seen at 1 h (p = 0.023, d = 0.25) and recovery by 24 h. Velocity in the fatigue condition was significantly lower than control at 15 min (p < 0.0005, d = 0.26) and 24 h (p = 0.031, d = 0.08) time points.

**Fig 4:**
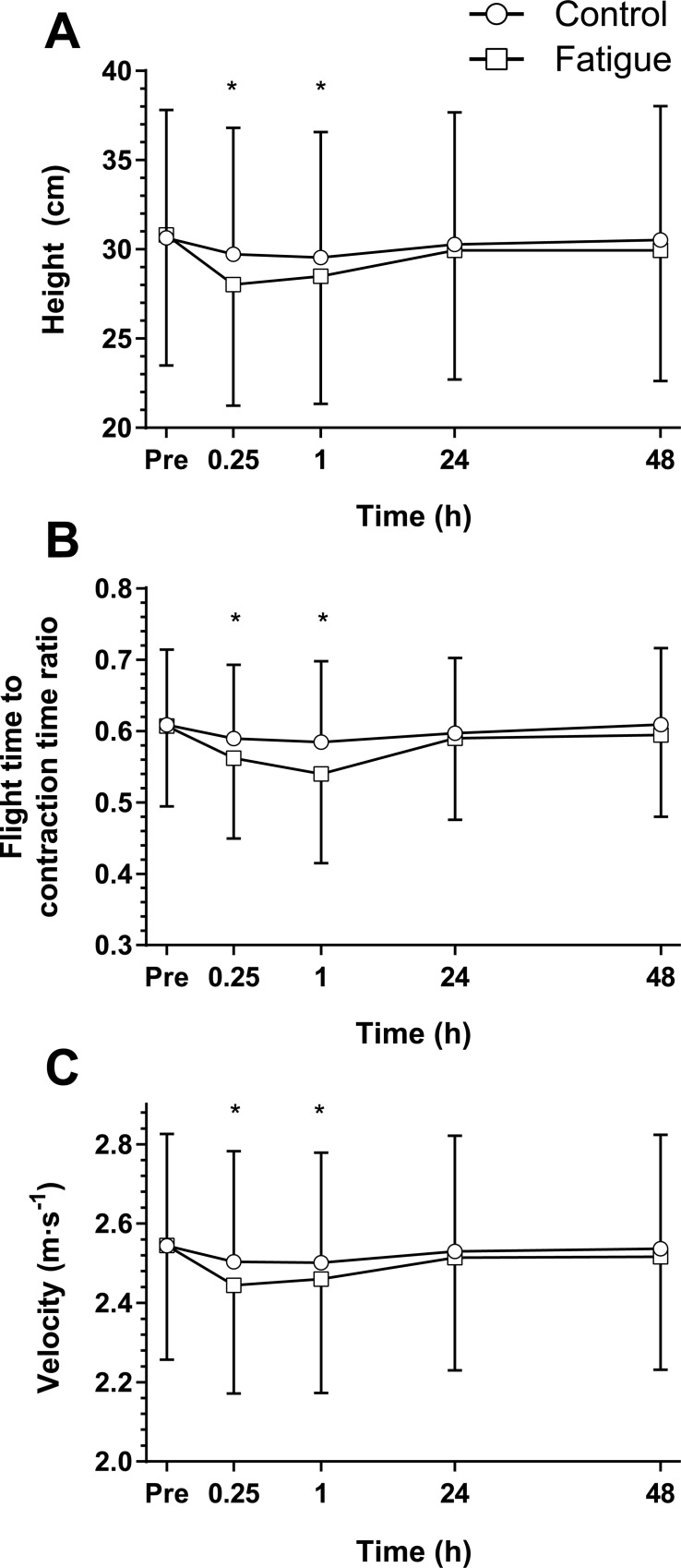
Counter movement jump results. The mean (SD) countermovement jump result (n = 31) for the control (circles) and fatigue interventions (squares) of (A) jump height; (B) flight time to contraction time ratio (FT:CT) and (C) peak propulsive (concentric) velocity. Time points from immediately prior (Pre) to 48 h post condition. *Significant (p < 0.05) difference between conditions at the identified time point using a Benjamini-Hochberg post hoc procedure.

Recorded CMJ velocity at baseline was 2.56 ± 0.27 m∙s^-1^ and 2.57 ± 0.26 m∙s^-1^ in control and fatigue respectively (Fig 4). A two-way interaction was seen between conditions (p = 0.005, d = 0.77). Control showed a peak decrease of 2% at 15 min (p < 0.0005, d = 0.14) and 1 h (p = 0.001, d = 0.15) post condition while fatigue had a peak decrease of 4% at 15 min (p < 0.0005, d = 0.35) and 1 h (p < 0.0005, d = 0.29) post intervention with a return to baseline by 24 h. CMJ Velocity was significantly lower in the fatigue condition at 15 min (p < 0.0005, d = 0.21) and 1 h (p = 0.005, d = 0.15) time points.

Baseline CMJ height was 30.63 ± 7.17 cm and 30.80 ± 7.32 cm in control and fatigue respectively. A two way interaction was shown (p = 0.001, d = 0.87) with the control condition significantly decreased by 3% at 15 min (p < 0.0005, d = 0.13) and 4% at 1 h (p = 0.010, d = 0.15) time points with a return by 24 h. Similarly, fatigue recorded a peak decrease in CMJ jump height of 9% at 15 min (p < 0.0005, d = 0.39) post intervention remaining 8% depressed at 1 h (p < 0.0005, d = 0.32) before returning to baseline at 24 h. A greater decrease in performance after the fatigue intervention resulted in significantly lower CMJ jump height in the fatigue condition at 15 min (p < 0.0005, d = 0.25) and 1 h (p = 0.008, d = 0.15) time points.

At baseline the CMJ FT:CT ratio was 0.61 ± 0.10 and 0.61 ± 0.11 for control and fatigue respectively, with a significant two-way interaction observed (p = 0.045, d = 0.63) over time. While no significant changes were observed in control over time, significant decreases of 8% and 11% were recorded at 15 min (p < 0.0005, d = 0.41) and 1 h (p = 0.002, d = 0.57) post fatigue intervention with a return to baseline at 24 h in the fatigue condition. When compared to control, fatigue condition results were significantly lower at the 15 min (p = 0.014, d = 0.25) and 1 h (p = 0.031, d = 0.38) time points.

The baseline control and fatigue condition SJ FT:CT ratios were 1.15 ± 0.24 and 1.21 ± 0.27 respectively. A two-way interaction was seen between condition and time for SJ FT:CT (p = 0.002, d = 0.77). No significant differences were seen across time in the control condition while fatigue showed a significant decrease of 6% at 1 h (p = 0.040, d = 0.25) post fatigue intervention with no significant difference at other time points. Additionally no significant differences between control and fatigue were seen at any time points.

**Fig 5:**
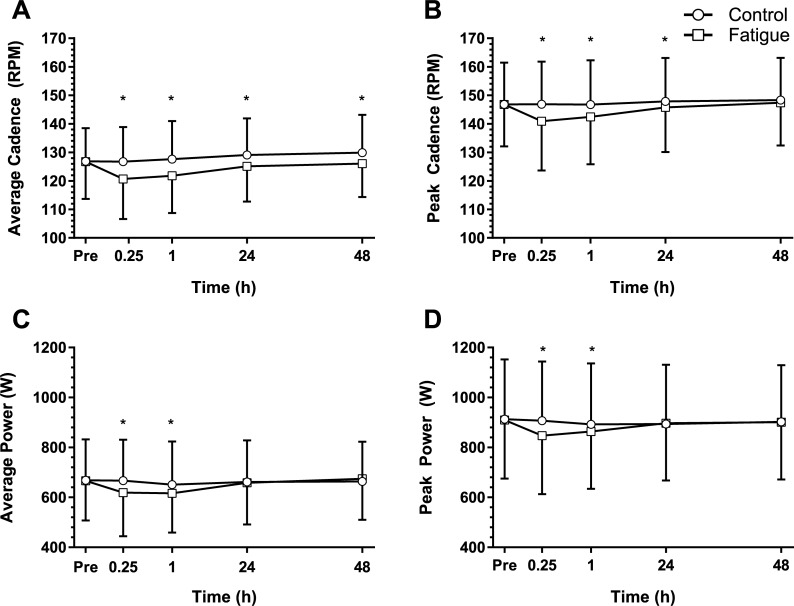
Sprint^max^ results. The mean (SD) maximal cycling sprint (Sprint^max^) result (n = 30) for the control (circles) and fatigue interventions (squares) of (A) sprint average cadence, (B) sprint peak cadence, (C) sprint average power and (D) sprint peak power variables. Time points from immediately prior (Pre) to 48 h post condition. *Significant (p < 0.05) difference between interventions at the identified time point using a Benjamini-Hochberg post hoc procedure.

The baseline average cadence observed in Sprint^max^ was 127 ± 12 RPM and 127±13 RPM in control and fatigue respectively (Fig 5) and a two-way interaction was observed (p = 0.003, d = 0.87). No significant differences were observed between baseline and later time points in the control condition. The fatigue condition resulted in a peak decrement of 5% at 15 min (p = 0.001, d = 0.45) post intervention with decrements of 4% seen at 1 h (p = 0.001, d = 0.38) post intervention. Fatigue was significantly lower than control at 15 min (p < 0.0005, d = 0.46), 1 h (p < 0.0005, d = 0.47), 24 h (p = 0.002, d = 0.34) and 48 h (p < 0.0005, d = 0.34) time points.

Peak cadence recorded during Sprint^max^ at baseline was 147 ± 15 RPM and 147 ± 15 RPM in control and fatigue respectively. A two-way interaction was seen (p < 0.0005, d = 1.22). No difference was seen in control time points while post intervention changes in the fatigue condition showed a peak 4% reduction at 15 min (p < 0.0005, d = 0.37) and a 3% decrease at 1 h (p < 0.0005, d = 0.28). Fatigue was significantly lower than control at 15 min (p < 0.0005, d = 0.36), 1 h (p < 0.0005, d = 0.27) and 24 h (p = 0.008, d = 0.14) time points.

Baseline Sprint^max^ peak power was 913 ± 240 W and 909 ± 235 W in control and fatigue respectively. A two-way interaction was seen in peak power (p = 0.002, d = 0.81). Peak power was not significantly different at any time points in control. Post intervention changes were seen in the fatigue condition with a significant decrease of 7% at 15 min (p < 0.0005, d = 0.27) and 5% at 1 h (p < 0.0005, d = 0.20). Fatigue was significantly lower than control at 15 min (p < 0.0005, d = 0.26) and 1 h (p = 0.041, d = 0.12) time points.

The average power recorded for Sprint^max^ at baseline was 669 ± 164 W and 666 ± 158 W for control and fatigue respectively. A two-way interaction was seen in average power (p < 0.0005, d = 0.97). No difference was seen in control time points, while the fatigue intervention had a significant decrease of 7% at both 15 min (p = 0.001, d = 0.28) and 1 h (p < 0.0005, d = 0.32) time points before returning to baseline. Average power in the fatigue condition was significantly lower than control at 15 min (p < 0.0005, d = 0.28) and 1 h (p = 0.004, d = 0.20) time points.

Though there was no difference between the control and fatigue Sprint^max^ average power output at 24 and 48 h time points, stepwise regressions were run to assess the ability of variables to explain power output at these time points. After removal of correlated variables the independent variables of the 24 h regression were 1 h NFQ, 1 h CMJ FT:CT, 1 h CMJ height and 1 h, SJ FTCT. Independent variables of the 48 h regression were 1 h and 48 h NFQ, 1 h and 48 h CMJ FT:CT, 1 h CMJ height and 1 h SJ FT:CT. Sprint^max^ average power output was used as the dependent variable. Due to the real world impracticality of daily measurement, variables taken at the 24 h time point were excluded from the 48 h stepwise regression. Additionally, where correlations between 15 min and 1 h variables were evident, the 1 h variable was retained in the regression. This resulted in variables from the 1 h time point analysed in the 24 h stepwise regression and variables from the 1 h and 48 h time points analysed in the 48 h stepwise regression.

The adjusted R^2^ for the 48 h model explained 51.7% of the variance (d = 2.22), a large effect size. CMJ height at 48 h explained 48 h Sprint^max^ average power output (p < .0005) with a regression equation of y = 187.618 + (1651.121 x 1 h CMJ height). In the 24 h stepwise regression the adjusted R^2^ for the overall model was 53.2% (d = 2.34), a large effect size. CMJ height at 1 h significantly explained 24 h average power output (p < .0005) with a regression equation of y = 165.905 + (1696.978 x 1 h CMJ height).

## Discussion

Our purpose was to assess whether a range of individual performance tests would be suitably sensitive to detect changes in Sprint^max^ performance up to 48 h after a fatigue inducing high intensity sprint cycle protocol and to assess whether individually or as a battery, these performance markers would explain the reduced performance in Sprint^max^ at these time points. This analysis was conducted with the intent of possibly creating a simple and practically relevant tool that could be used to determine when an athlete or a patient was ready to cope with their next training (exercise) stimulus.

We identified Sprint^max^, NFQ, CMJ and SJ as performance tests that changed in response to a high intensity sprint cycling fatigue protocol. As predicted, Sprint^max^ variables showed decrements up to 48 h following fatigue inducement however effect sizes were small. Interestingly the subjective NFQ score was the most sensitive to fatigue with medium to large effect sizes for changes occurring up to 24 h post fatigue. Although sex differences may play a role in NFQ responses, similar perceptions of soreness and recovery have been reported between males and females [23]. While CMJ jump height, velocity and FT:CT responded to fatigue this was only in the short term (up to 1 h post) with small and trivial effect sizes. In contrast to this in the SJ variable, only velocity was indicative of fatigue 15 min and 24 h after the high intensity sprint cycle protocol and even then, only with small and trivial effect sizes. Though no change was observed between fatigue and control power outputs at 24 and 48 h, stepwise regression analysis suggests that the a single marker, CMJ height, explained 53.2% and 51.7% of Sprint^max^ power output at these time points.

Although Sprint^max^ average power was only reduced 15 min and 1 h following the intervention protocol, average cadence remained lower in the fatigue condition 24 h and 48 h post with reductions also present in peak cadence 24 h after. The reduced cadence in the absence of power decrements, should theoretically require a greater force production per pedal stroke to achieve the given power output. Typically, the requirement of competition is to produce the highest average power output, making this cadence reduction practically insignificant, however completing Sprint^max^ at a non-preferred cadence suggests participants were still feeling the effects of fatigue which may have manifested in perceptual feeling of fatigue seen in the NFQ at later time points. The inability to reproduce higher cadences may be a result of reductions in contraction velocities or slowing of muscle relaxation [24]. A slowed relaxation rate could affect cycling due to concentric leg contractions occurring before relaxation of the opposing leg therefore affecting peak pedal rate and optimal pedal cadences [25]. However further investigations that record direct pedal forces are required to confirm this hypothesis.

Perceptual questionnaires have previously been used to monitor acute and chronic training periods [13, 26–28]. Consistent with previous research [13, 26, 27, 29] this neuromuscular fatigue questionnaire was sensitive to acute fatigue with significant differences between conditions seen up to 24 h post. As reported previously [30], the change in perception of fatigue was not directly matched to changes in performance with an increase in perceived fatigue at 24 h despite an absence of power decrements. Reductions in Sprint^max^ cadence were observed at 24 and 48 h however, and may have been reflected in perceptual fatigue changes at later time points. Psychological motivation has been shown to play a role in performance under fatigued conditions with threefold higher power production recorded in brief cycle sprints immediately after voluntary exhaustion [31]. The possibility of participant motivation influencing these observations should not be discounted due to the brief test duration of multiple performance markers in this research.

The results of the present study are consistent with published research suggesting that vertical jump variables are sensitive to sprint cycling fatigue [6] with CMJ variables significantly reduced 1 h post intervention and a reduction in SJ peak velocity seen at 15 min and 24 h post but not 1 h post. However, the degree of sensitivity of CMJ and SJ tests is likely dependent on the magnitude and type of fatigue inducement with little change seen in jump variables beyond 24 h post intervention. The slight differences observed between CMJ and SJ variables may be reflective of the different metabolic contractile conditions of the high intensity sprint cycling exercise bout and/or further evidence of changes in contraction velocities or slowing of muscle relaxation processes [24]. The complexity of the CMJ movement results in movement mechanics not seen in the SJ. A greater uptake of muscle slack and increased build-up of stimulation during the countermovement is thought to be a main contributor [32] to the greater amount of work completed in the CMJ. It is possible that differences in movement mechanics or the difference in total work of the two jump types may play a role in the sensitivity of the two tests. The lack of decrements in both jump and Sprint^max^ power variables at time periods longer than 24 h suggests that multiple sprint cycling protocols do not produce long lasting changes in explosive lower body power production. Conversely, an exercise intervention utilising greater eccentric muscle contraction may have displayed a greater fatigue response due to the fact that eccentric exercise modalities result in substantial changes to the neuromuscular system [33]. Changes such as increased muscle microlesions [33] and resultant increased soreness, is likely to affect perceptual fatigue as well as the ability to perform physical performance tests like those utilised in this study. This is supported by reported CMJ and SJ decrements after unaccustomed eccentric cycling that were not observed in concentric cycling [34] as well as decrements in CMJ and SJ variables for team sports requiring greater eccentric contractions [35–38] The modality used to induce fatigue in this study may have resulted in less muscle damage due to the low volume, yet high intensity and lack of eccentric muscle contraction. Though CMJ and SJ performance differences were seen, changes at 1 h post intervention were not practically useful for coaches and exercise scientists when considering an investigation outcome was to provide insight on whether the athlete was ready for their next stimulus.

The fatigue test battery showed no difference between 24 and 48 h variables to explain power output with 53.2% versus 51.7% respectively. A single variable, CMJ height, explained Sprint^max^ average power output at both time points. This was likely affected by auto correlations between potential variables however with only 4 and 6 variables entered in 24 and 48 h regressions. Interestingly, despite having medium to large effect sizes the NFQ did not explain power output at any time point. Finally the lack of Sprint^max^ changes observed at 24 h and 48 h reduced the ability of the stepwise regression to assess fatigue at these time points.

Of the six performance tests completed, the Stroop task, postural sway and IMTP did not significantly differ between the control and fatigue conditions. Psychomotor assessments such as the Stroop task have been proposed as a measure of fatigue [39] however we observed no significant differences. Differences in participant digestion rates post feeding may have affected cognitive performance in the Stroop task due to the effect that blood glucose levels have on cognitive ability [40]. Additionally, the duration of fatigue inducement may have been too brief with cognitive decrements observed in longer overreaching protocols [13, 41, 42].

Despite reported decrements in postural control after cycling protocols [43–45], none were seen in the present study. Differing from previous studies [4, 44, 46, 47], this research assessed sway >15 min after fatigue inducement as our assessment time frames required relevance to making practically informative decisions, yet these assessment times potentially affected the sensitivity of this variable.

Though use of the IMTP as a performance metric exists [2], this study is one of few to utilise the test as a performance metric after fatigue inducement. Consistent with these studies, no decrements in IMTP performance were seen after fatigue [48, 49]. The lack of sensitivity of the IMTP as a gross muscle action task to fatigue implicates the possibility of multiple motor control strategies that are able to produce near maximal contractions when only short contraction times are required. We would argue that this notion of multiple near maximal control strategy is supported by the reports that IMTP maximal force change is a worthwhile training adaptation metric in team sport and individual athletes [50–52].

Though a large number of participants completed this study, a heterogeneous cohort was tested and may have affected statistical power. Additionally, the post control-intervention warmup may not have provided sufficient preparation possibly explaining decrements seen at the 15 min time point after the control intervention. An effort was made to reduce the learning effect by completion of an extensive familiarisation however consistent with previous research a learning effect was seen in the Stroop task. Finally the lack of power decrement seen in Sprint^max^ at 24 and 48 h reduced the applicability of 24 and 48 h test battery data to assess fatigue despite the reduction in cadence at these time points suggested fatigue was still present. Further research is needed on fatigue inducement employing different muscle contractile conditions (i.e. stretch-shortening cycle inclusive) that result in performance decrements at >24 h. This may elucidate whether battery variables respond at these time points. Additionally more research is needed on the fatigue test battery across an overreaching phase to determine if sensitivity of variables increases with accumulation of fatigue.

This research reports that NFQ and CMJ jump variables such as height, velocity and FT:CT are suitable to monitor fatigue at multiple time points after sprint cycling fatigue, while using CMJ height explains >51% of sprint power output. The results suggest that a maximal intensity sprint cycle protocol consisting of four sets of 10 x 6 s sprints may prove a useful high intensity low volume training stimulus due to small amounts of fatigue seen at >1 h.

## Supporting information

S1 File Supplementary fatigue test battery data. Combined results of all fatigue test battery tests.
(XLSX)

S2 File Neuromuscular fatigue questionnaire. Adapted fatigue questionnaire.
(PDF)
