# Supplementary material for: The use of a functional test battery as a non-invasive method of fatigue assessment
Source: PLoS One. 2019 Feb 28;14(2):e0212870. doi: 10.1371/journal.pone.0212870 (PMC6394954; doi:10.1371/journal.pone.0212870)
Supplement: S2 File — Adapted fatigue questionnaire. (PDF) [file pone.0212870.s002.pdf]

Article Name
The use of a functional test battery as a non-invasive method of fatigue assessment

Journal Name
PLOS ONE

Author Names
Steven Hughes, Dale W Chapman, Guy G Haff, Sophia Nimphius

Corresponding Author
Steven Hughes
Physiology Discipline, Australian Institute of Sport
Centre for Exercise and Sports Science Research, School of Medical and Health Sciences, Edith Cowan University

Address
Physiology Department
Australian Institute of Sport
PO Box 176 Leverrier Street, Belconnen
Canberra, Australia Capital Territory, 2616
Australia
Tel: (61) 4 10 726 872
Email: shug5877@uni.sydney.edu.au

Neuromuscular Fatigue Questionnaire

Please circle one response for each of the following questions.

1) Are you currently experiencing an increased level of muscular fatigue?

Not at all	Slightly	A fair amount	A moderate amount	A considerable amount	A lot	An Extreme amount
0	1	2	3	4	5	6

2) Is your muscle fatigue causing you difficulty in performing everyday activities? For example: walking up/ down stairs, standing to sitting/ sitting to standing, putting on shoes etc.

Not at all	Slightly	A fair amount	A moderate amount	A considerable amount	A lot	An Extreme amount
0	1	2	3	4	5	6

3) Is your muscular fatigue causing you an increased level of pain?

Not at all	Slightly	A fair amount	A moderate amount	A considerable amount	A lot	An Extreme amount
0	1	2	3	4	5	6

4) Do you feel an increased level of tiredness/general fatigue as a result of your muscular fatigue?

Not at all	Slightly	A fair amount	A moderate amount	A considerable amount	A lot	An Extreme amount
0	1	2	3	4	5	6
